# Supplementary material for: A comparative analysis of toxin gene families across diverse sea anemone species
Source: Toxicon X. 2025 Mar 7;26:100217. doi: 10.1016/j.toxcx.2025.100217 (PMC11952004; doi:10.1016/j.toxcx.2025.100217)
Supplement: Multimedia component 2 [file mmc2.docx]

**A comparative analysis of toxin gene families across diverse sea anemone species**

**Hayden L. Smith ^1^, Daniel A. Broszczak ^2^, Chloe A. van der Burg ^3^, Joachim M. Surm ^4^, Libby Liggins ^5^, Raymond S. Norton ^6,7^ and Peter J. Prentis ^1,8,*^**

1 School of Biology and Environmental Sciences, Science and Engineering Faculty, Queensland University of Technology, Brisbane, Australia

2 School of Biomedical Sciences, Faculty of Health, Queensland University of Technology, Brisbane 4000, Australia

3 Department of Anatomy, School of Biomedical Sciences, University of Otago, Dunedin 9016, New Zealand

4 Faculty of Biology, Ludwig-Maximilians-Universität Munich, Munich, D-80539, Germany

5 School of Biological Sciences, University of Auckland, Auckland 1010, New Zealand

6 Medicinal Chemistry, Monash Institute of Pharmaceutical Sciences, Monash University, Parkville, Victoria 3052, Australia

7 ARC Centre for Fragment-Based Design, Monash University, Parkville, Victoria, 3052, Australia

8 Centre for Agriculture and the Bioeconomy, Queensland University of Technology, Brisbane, Australia

* Corresponding Author: p.prentis@qut.edu.au


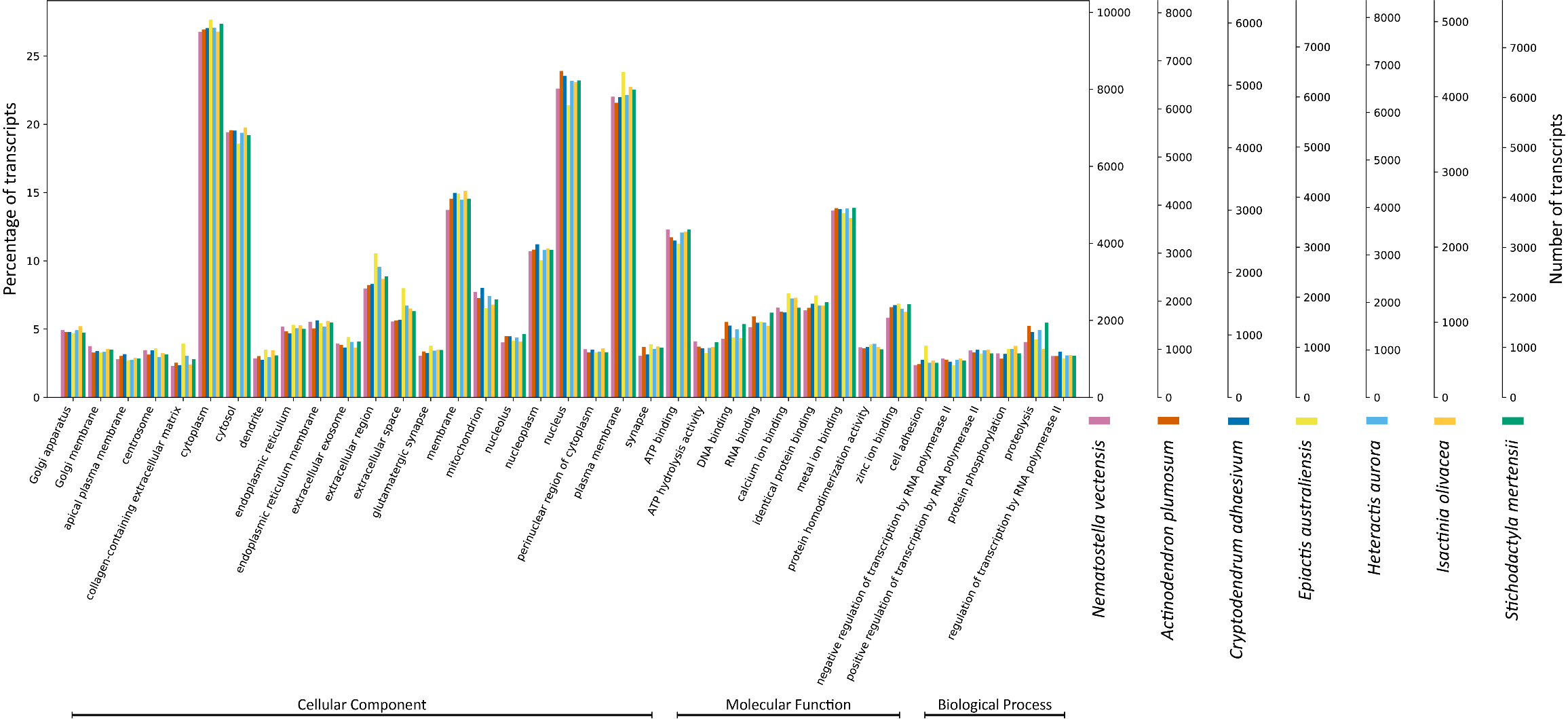


Supplementary Figure S1: Percentage of transcripts with known gene ontology terms for the six transcriptomes generated in this study. To consolidate visual output, only values for gene ontology terms with copy numbers greater than 1,000 transcripts are shown.


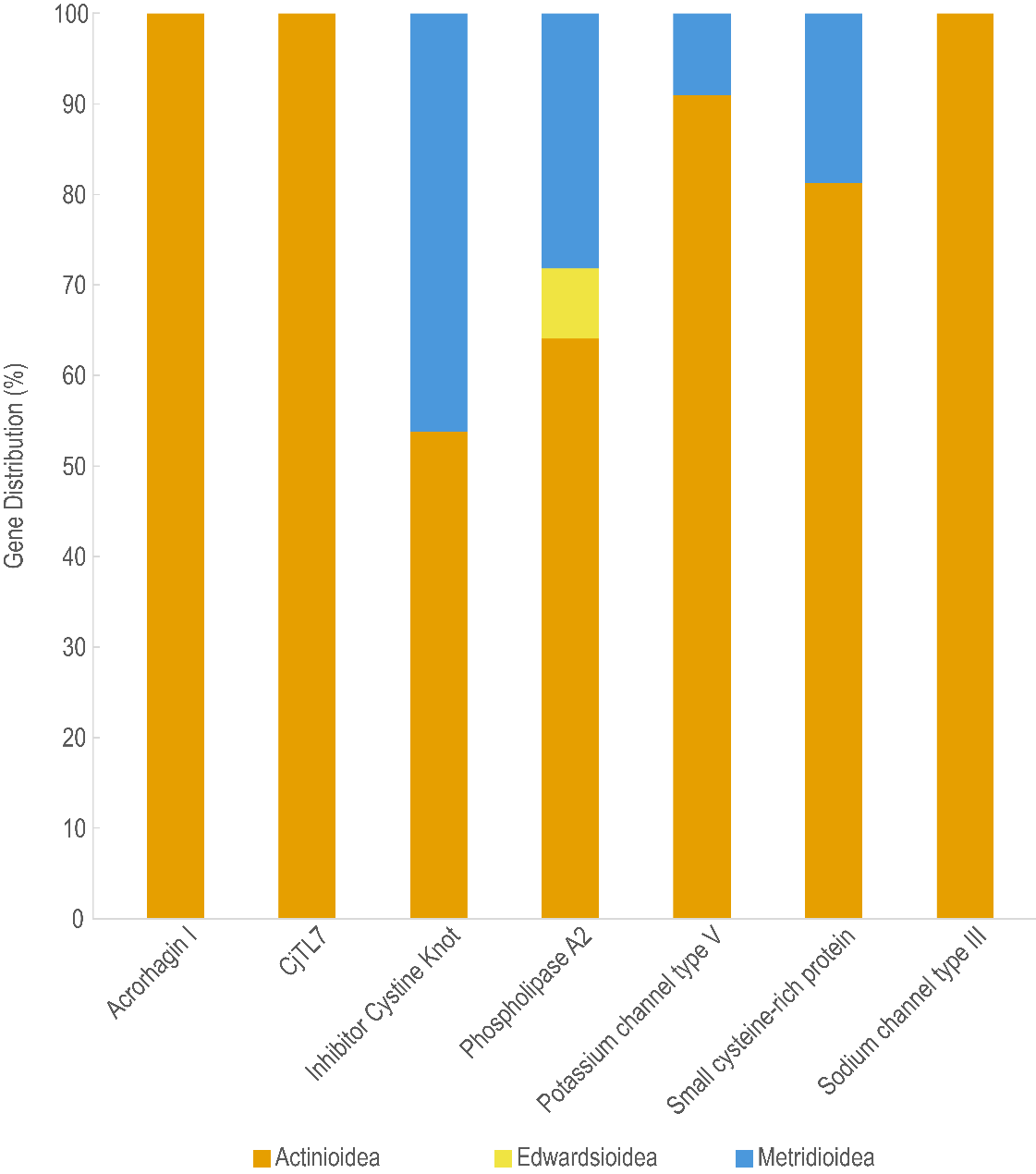


Supplementary Figure S2: Percent distribution of gene copy numbers for seven toxins of interest across three superfamilies of sea anemones, Actinioidea, Edwardsioidea and Metridioidea.


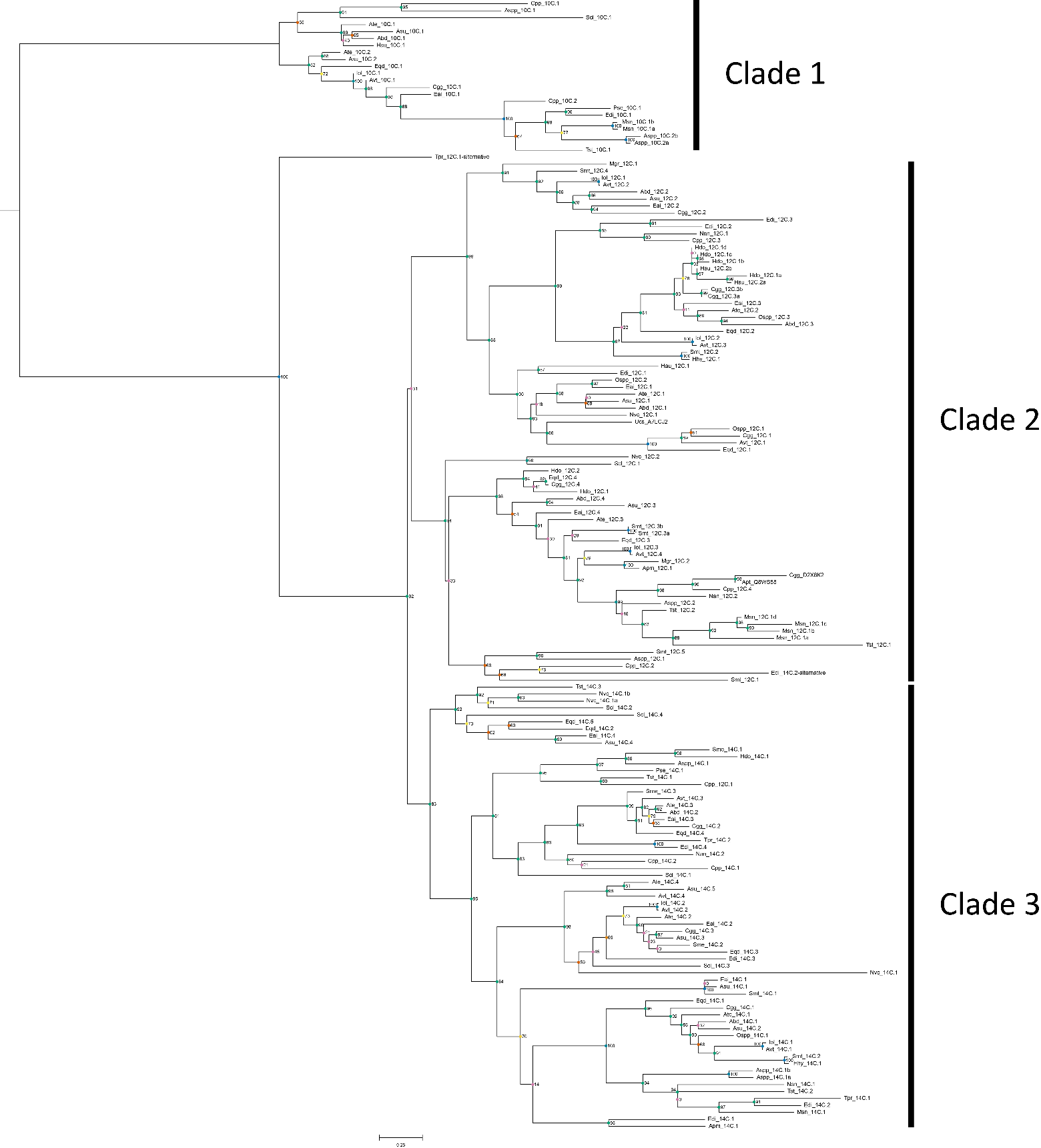


Supplementary Figure S3: Maximum-likelihood phylogenetic tree of candidate sequences for the phospholipase A2 toxin family. Support values shown as Maximum Likelihood bootstrap (0-100). Gene transcripts were identified based on their species name followed by count number or isoform designation denoted with alphanumeric values. Sequences denoted with a six-digit alphanumeric code are from the Swiss-Prot/UniProt databases.


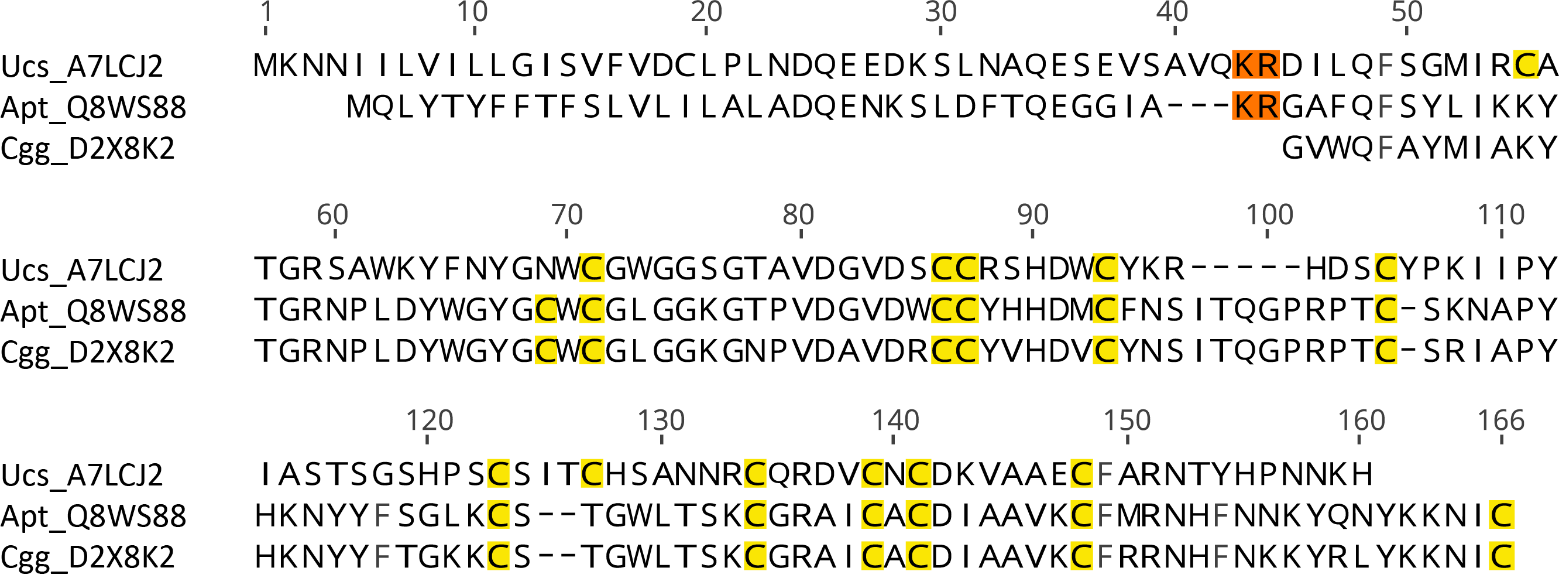


Supplementary Figure S4: Alignment of characterised 12-cysteine phospholipase A2 sequences from UniProt showing two cysteine framework structures, with highlighted sections for cysteine (C) residues and known cleavage site motifs (KR). Abbreviations: *Urticina crassicornis* (Ucs), *Calliactis palliata* (Apt; formerly *Adamsia palliata*), *Condylactis gigantea* (Cgg). Sequences denoted with a six-digit alphanumeric code from the Swiss-Prot/UniProt databases.


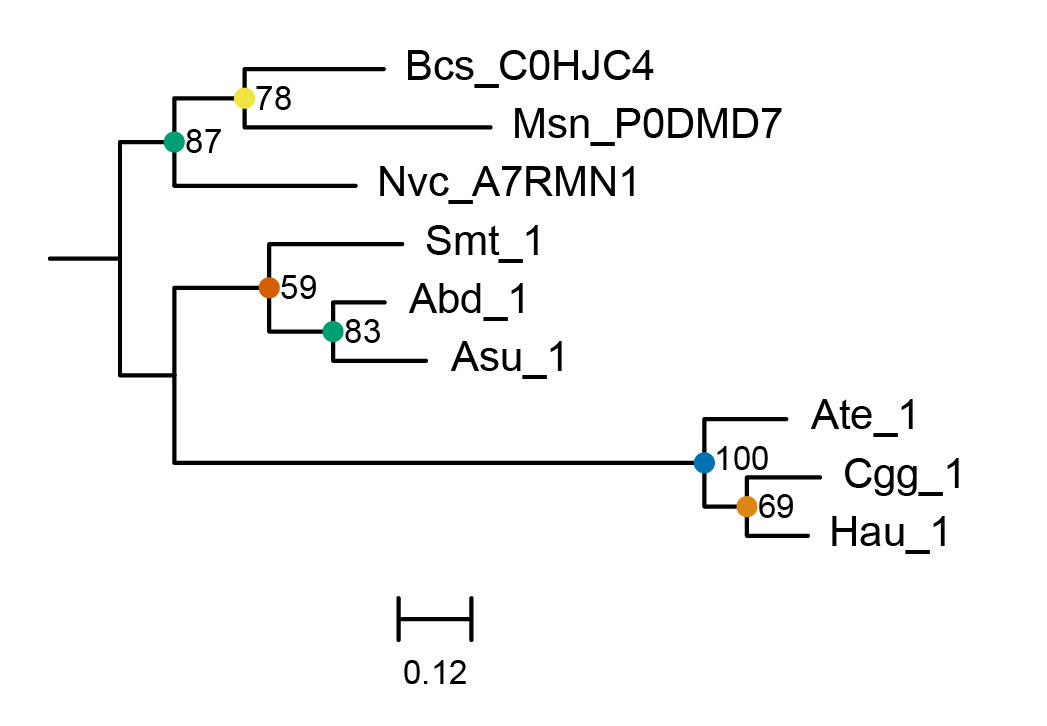


Supplementary Figure S5: Maximum-likelihood phylogenetic tree for the potassium channel toxin type V family sequences derived from Clade 1 and Clade 2 of Figure 6 in Manuscript. Support values shown as Maximum Likelihood bootstrap (0-100). Gene transcripts were identified based on their species name followed by count number or isoform designation denoted with alphanumeric values. Sequences denoted with a six-digit alphanumeric code are from the Swiss-Prot/UniProt databases.
